# Probiotics in Periodontal Diseases: Mechanisms, Evidence Mapping, Limitations, and Future Directions

**DOI:** 10.7759/cureus.96042

**Published:** 2025-11-03

**Authors:** Hamza Alasbily, Huda H Mohamed, Adnan Asheibi, Manal S Bazina, Ali Alkaseh, Hana M Ghaith, Fardous Ali Fahmi

**Affiliations:** 1 Basic Medical Science, University of Benghazi, Benghazi, LBY; 2 Periodontology, Faculty of Dentistry, University of Benghazi, Benghazi, LBY; 3 Medicine, Libyan International University, Benghazi, LBY; 4 General Dentistry, Faculty of Dentistry, University of Benghazi, Benghazi, LBY; 5 Biological Sciences, Libyan International University, Benghazi, LBY

**Keywords:** alveolar bone loss, clinical attachment level, periodontal disease, probing depth, probiotics

## Abstract

Periodontal disease represents a spectrum of inflammatory disorders that impact the teeth's supporting tissues. It is initiated by the buildup of microbial plaque and sustained by dysbiosis, an imbalance in the oral microbiome that causes tissue damage and disturbs host-microbe homeostasis. These diseases can range from reversible inflammation of the gingiva (gingivitis) to irreversible destruction of the periodontal apparatus (periodontitis).

While scaling and root planing, with or without antimicrobials, can effectively reduce bacterial burden, mechanical debridement by itself may not restore microbial symbiosis and may allow disease-associated microbial populations to persist. Incomplete pathogen clearance from deep pockets, residual calculus, or inaccessible root surfaces frequently results in bacterial regrowth and disease progression.

Probiotics have emerged as a possible alternative or supplement in periodontal therapy. Their possible benefits include microbial balance restoration in the oral cavity, as well as anti-inflammatory, immunomodulatory, and bone-preserving actions.

Nonetheless, the strain-specific effects, dosage regimen, safety profile especially in certain patients and the absence of large-scale, long‑term randomized controlled trials to definitively establish their efficacy remain as concerns. This review discusses the mechanisms through which probiotics may influence periodontal diseases, systematically maps preclinical and clinical evidence, and highlights current limitations and future directions for their application in periodontal therapy.

## Introduction and background

Periodontal diseases are chronic inflammatory conditions initiated by a dysbiotic dental biofilm and mediated by an immune response affecting the supporting periodontal tissues. Disease progression and severity are primarily determined by risk factors, including environmental and genetic susceptibility [1,2]. Periodontal health is maintained by a balanced microbial community; disruption of this equilibrium leads to microbiota-host dysbiosis [3,4]. The pathogenic sequence begins when Gram-positive and Gram-negative bacteria attach, grow, and colonize the tooth surface, eventually extending subgingivally [5]. These conditions favor the proliferation of anaerobic species, particularly those of the orange and red complexes, whose collagenases and proteases trigger inflammatory responses and contribute to periodontal tissue degradation [6,7].

Although conventional therapies such as scaling and root planing (SRP), with adjunctive antimicrobials, reduce bacterial load, they do not effectively restore the balance of the oral microbiome, and broad-spectrum antimicrobials may further disturb microbial homeostasis [8-10]. Consequently, novel approaches such as probiotics are being explored for their potential to modulate microbial ecology and support periodontal health.

Probiotics are live microorganisms that have demonstrated encouraging results in dentistry when given in sufficient doses. By promoting immunological function, lowering inflammation, and maintaining microbial balance, they improve general health. Species such as *Lactobacillus*, *Bifidobacterium*, *Streptococcus*, and *Saccharomyces* can help maintain the balance of oral microbes., reduce inflammation, and modulate immune responses [11]. As a result, probiotics are emerging as potential adjuvants to conventional periodontal therapies [12]. This review discusses the mechanisms through which probiotics may influence periodontal diseases, systematically maps preclinical and clinical evidence, and highlights current limitations and future directions for their application in periodontal therapy.

Data source 

We performed a comprehensive search using multiple databases (PubMed, Scopus, Web of Science, Directory of Open Access Journals (DOAJ), and Google Scholar) and publishing platforms (Science Direct, Wiley Online Library, Springer Link, Taylor & Francis Online, and Multidisciplinary Digital Publishing Institute (MDPI)), where only peer-reviewed articles published in English were included. The search focused on studies published from 2018 onward and was conducted using a combination of Medical Subject Heading (MeSH) terms - “Probiotics,” “Periodontal Diseases,” “Periodontitis,” “Alveolar Bone Loss,” and “Microbiota” - together with relevant free-text keywords. Non-peer-reviewed publications and non-English articles were excluded.

## Review

Mechanisms of probiotics in periodontal diseases

Antimicrobial Effects

Probiotics exert a notable antimicrobial activity against various microorganisms, including oral pathogens (*Porphyromonas gingivalis*, *Streptococcus mutans*, *Tannerella forsythia*, and *Candida albicans*), through various mechanisms, including competitive exclusion, production of antimicrobial compounds, and quorum quenching (QQ), which refers to interfering with bacterial quorum sensing - a communication mechanism used by bacteria to coordinate behavior in response to population density [13-15]. In competitive exclusion, probiotics inhibit the pathogens by competing for nutrients and adhesion sites, where they strongly adhere to mucosal surfaces, a trait that is influenced by bacterial surface hydrophobicity, thus reducing the access of pathogens to host receptors [16,17] and providing a physical block against the adherence of pathogens [13,14,16,18]. Another antimicrobial mechanism of probiotics is their ability to produce molecules with antibacterial effects such as bacteriocins, lactic acid, and hydrogen peroxide. Bacteriocins can interfere with nucleic acid replication and protein synthesis and induce pore formation within pathogen cell membrane, which subsequently leads to a leakage of cellular contents [13,19]. Additionally, probiotics can disrupt bacterial communication systems essential for virulence, where they produce enzymes or inhibitors of QQ signaling and degrade signaling molecules like acylated homoserine lactones (AHLs) or block their receptors and subsequently interfere with pathogens' coordination and biofilm formation [20-22].

 *Anti-inflammatory and Immune Modulation*

The anti-inflammatory and immunomodulatory effects of probiotics involve several mechanisms, many of which are strain-specific, including the modulation of cytokines, toll-like receptors (TLRs), nuclear factor kappa-light-chain-enhancer of activated B cells (NF-κB), regulatory T cells (Tregs), and histamine signaling, collectively contributing to the reduction of the inflammatory responses.

The anti-inflammatory properties of probiotics are primarily mediated through influencing cytokines where they suppress pro-inflammatory cytokines such as interleukin (IL)-1β, IL-6, tumor necrosis factor-alpha (TNF-α), and matrix metalloproteinase-8 (MMP-8), while enhancing anti-inflammatory cytokines like IL-10 and tissue inhibitor of metalloproteinase-1 (TIMP-1) where this modulation of cytokines helps balance inflammation [23-31]. TLR modulation also plays an essential role in prebiotic-mediated immune regulation where specific probiotics interact with TLRs expressed on sentinel immune cells including macrophages and dendritic cells. For instance, probiotics can inhibit the lipopolysaccharide binding site for TLR 2 and TLR4, thus attenuating inflammatory responses mediated by NF-κB activation [25,32]. TLR9 detects bacterial DNA-containing unmethylated CpG motifs released by probiotics, which promotes an anti-inflammatory response through Treg cell activation. Interestingly, TLR10 is unique among TLRs for its inhibitory effect on NF-κB-mediated responses when co-expressed with other TLRs such as TLR1/TLR2 or TLR2/TLR6 [31,33].

The NF-κB pathway is another target for probiotic actions. Through this pathway, probiotics promote the inhibition of IκBα degradation and the suppression of phosphorylation and nuclear transcriptional activity of NF-κB subunits like p65, thereby reducing the production of pro-inflammatory cytokines [23,25,34,35]. An additional mechanism of probiotics' anti-inflammatory actions is the regulation of Tregs, which are essential mediators of immune tolerance and inflammation suppression. Enhancing Treg differentiation through stimulating dendritic cells to release cytokines like IL-10 and transforming growth factor-beta (TGF-β) leads to the promotion of naïve T cell maturation into Tregs, which subsequently suppress pro-inflammatory Th17 polarization, shifting toward an anti-inflammatory profile and helping to modulate the immune response [23,36]. The modulation of histamine further contributes to probiotics' anti-inflammatory actions where specific probiotic strains are able to produce histamine, which interacts with histamine-2 receptors on antigen-presenting cells, leading to decreased production of pro-inflammatory markers like TNF-α, IL-12, and monocyte chemotactic protein-1, and supporting anti-inflammatory pathways [23].

Microenvironmental Acidity and Enamel Protection

Probiotics can help maintain balanced oral pH and protect enamel through several strain-specific pathways - both direct and indirect. A primary mechanism is the arginine deiminase system, where dietary arginine is converted to ammonia by certain probiotic strains, which then form ammonium ions. This process raises salivary pH, creating a more alkaline environment that counteracts the acids produced by cariogenic bacteria [37,38]. Probiotics also compete with acid-producing pathogens, reducing their prevalence, subsequently limiting acid production and helping to maintain more neutral pH in saliva [39]. Enamel protection is another action of probiotics, which is gained by counteracting demineralization processes. Through their ability to neutralize organic acids produced during bacterial fermentation, probiotics help preserve calcium and phosphate levels, which are essential for enamel integrity [40,41]. Owing to this buffering, enamel erosion is reduced, and the process of remineralization is supported under acidic challenges [40].

Anti-oxidant Effects

Through multiple mechanisms, probiotics exert antioxidant properties, many of which are strain-dependent. The production of antioxidant enzymes - superoxide dismutase (SOD), catalase, and glutathione peroxidase (GPx) - represents a direct mechanism by which probiotics scavenge reactive oxygen species (ROS) [42,43]. Additionally, the non-enzymatic metabolites produced by probiotics such as glutathione, folic acid, and short-chain fatty acids neutralize free radicals and reduce oxidative damage [42]. Exopolysaccharides secreted by certain strains also exhibit radical-scavenging activity by binding iron ions and preventing Fenton reactions that generate hydroxyl radicals [44,45]. Another proposed mechanism underlying the antioxidant effects of probiotics is the regulation of key cellular antioxidant pathways, including the nuclear factor erythroid 2-related factor 2-Kelch-like ECH-associated protein 1-antioxidant response element (Nrf2-Keap1-ARE) pathway. Activation of Nrf2 leads to its translocation into the nucleus, where it binds ARE and upregulates genes encoding antioxidant enzymes and detoxifying proteins. Through this mechanism, probiotics enhance cellular resilience against oxidative stress [42,43,46,47]. Furthermore, probiotics influence other signaling pathways that are involved in maintaining cellular integrity under oxidative conditions, such as mitogen-activated protein kinase (MAPK) and protein kinase C (PKC) [42,43,48].

In addition to the previously mentioned mechanisms, the antioxidant effects of probiotics extend to metal chelation and reduction of ROS generation. By chelation of transition metals like Fe^2+^ and Cu^2+^, probiotics effectively reduce their availability for radical generation. The chelation capacity of probiotics varies among strains but has been shown to significantly contribute to overall antioxidant potential [45,49]. Probiotics also exert the capability of inhibiting pro-oxidant enzymes such as nicotinamide adenine dinucleotide phosphate oxidase (NOX) and cyclooxygenase-2 (COX-2), thereby reducing ROS generation [48].

Bone Metabolism Modulation

Through immunomodulatory effects and enhancement of mineral absorption, probiotics influence bone metabolism. One important mechanism is the enrichment of butyrate-producing gut microbiota, which increases the production of short-chain fatty acids (SCFAs) that strengthen the intestinal barrier by regulating tight junction proteins and help suppress osteoclastogenic cytokines such as IL-6, IL-17, and IL-23. These immune modulations collectively contribute to decreased alveolar bone resorption [50,51]. Probiotics also regulate bone remodeling by modulating other key cytokines and signaling pathways involved in osteoclastogenesis. They reduce proinflammatory mediators, including IL-1β, TNF-α, and IL-6, while increasing osteoprotegerin (OPG), leading to the suppression of receptor activation of nuclear factor kappa B ligand (RANKL)-mediated osteoclast differentiation, shifting the RANKL/OPG ratio in favor of bone formation [51,52]. Mineral density is also enhanced by probiotics, which promote mineral absorption essential for bone formation, partly attributed to SCFA-mediated improvement in gut health [53]. Vitamin D metabolism has also been shown to be influenced by probiotics where supplementation was associated with elevated circulating 25-hydroxyvitamin D levels [54].

Periodontal Tissue Regeneration

**Figure 1:**
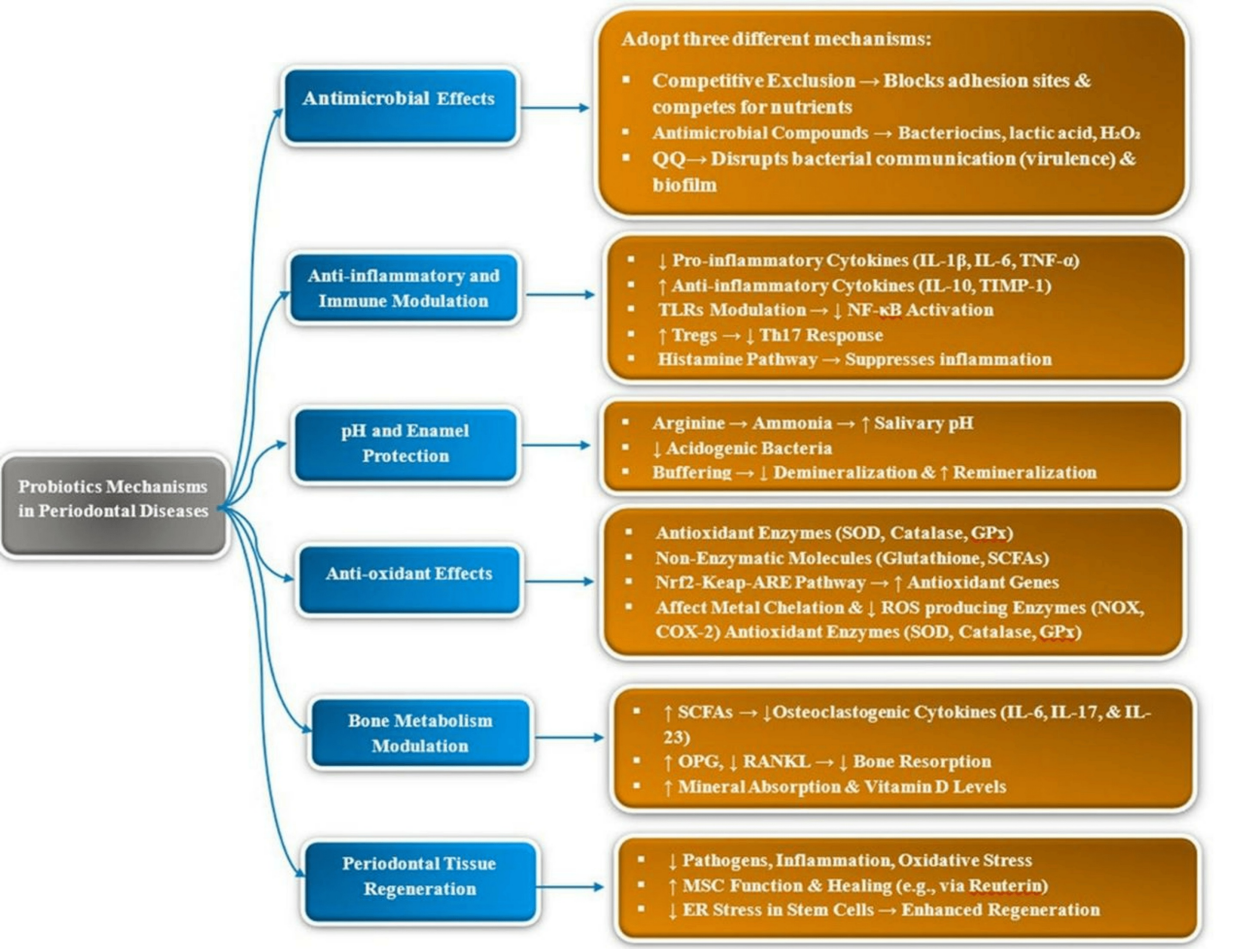
Summary of the mechanisms of probiotics in periodontal diseases Image credit: Created by the authors using Microsoft Word (version 16). Abbreviations: QQ, Quorum quenching; IL, interleukin; TNF-α, tumor necrosis factor-alpha; TIMP-1, tissue inhibitor of metalloproteinase-1; TLRs, Toll-like receptors; NF-κB, nuclear factor kappa-light-chain-enhancer of activated B cells; Tregs, regulatory T cells; Th17, T helper 17 cells; SOD, superoxide dismutase; GPx, glutathione peroxidase; SCFAs, short-chain fatty acids; Nrf2-Keap-ARE, nuclear factor.

Probiotics’ potential to enhance periodontal tissue regeneration is based on their ability to provide a healthy environment and restore the balance necessary for periodontal healing and regeneration through suppression of pathogenic bacteria, reduction of inflammation, neutralization of oxidative stress, stabilization of oral pH, and enhancement of bone metabolism. A primary example is reuterin extracted from *Limosilactobacillus reuteri*, which has been found to positively affect the function of mesenchymal stem cells (MSC) and enhance soft tissue healing. It also inhibits the endoplasmic reticulum (ER) stress in periodontal ligament stem cells and regulates intercellular pathways, which can facilitate the regeneration of periodontal tissue [55,56]. The proposed mechanisms of probiotics in periodontal diseases are summarized in Figure 1.

Preclinical evidence

**Figure 2:**
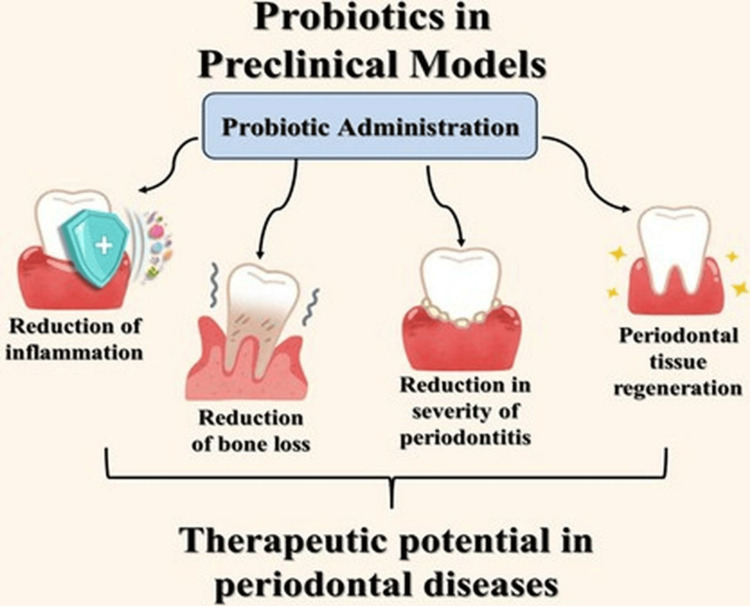
Potential benefits of probiotics in preclinical models. Image credit: Created by the authors using Canva software.

In several preclinical models, probiotics have demonstrated potential benefits in the context of periodontal diseases, through mechanisms involving the reduction of inflammation and bone loss as well as the enhancement of periodontal tissue regeneration, as shown in Figure 2.

Silva et al. evaluate the anti-hyperglycemic, anti-bone loss, and anti-inflammatory properties of *Lactobacillus rhamnosus* EM1107 in diabetic rats with ligature-induced periodontitis, alone and in combination with metformin. In this experiment, 114 male Wistar rats were assigned to six distinct groups: a control group, a group with induced periodontitis, one with both diabetes and periodontitis, a probiotics-only group, another receiving probiotics alongside diabetes, and a final group treated with probiotics, diabetes, and metformin. Diabetes was induced via streptozotocin injection, while periodontitis was established by placing ligatures around the teeth. Probiotics and metformin were administered orally through gavage for the duration of the study. Upon analyzing blood, gingival tissue, and jawbone samples, researchers observed notably lower levels of the inflammatory markers IL-1β and TNF-α in the groups that received probiotics. These groups also demonstrated improved glycemic control. Notably, the combination of probiotics and metformin led to a significant reduction in alveolar bone loss, as evidenced by both histological assessments and micro-computed tomography (micro-CT) scans. In addition, a decrease in the antioxidant enzymes SOD and GPx was detected in the probiotic-treated groups. These findings suggest that *L. rhamnosus* EM1107, whether used alone or in conjunction with metformin, may help modulate immune responses and offer protection against periodontal destruction under diabetic conditions [57].

The effects of systemic and local probiotic administration on alveolar bone loss in a rat model of metabolic syndrome-associated periodontitis were investigated by Moreira et al. In the study, 48 rats were fed a high-fat diet for 16 weeks to induce metabolic syndrome (MS) and then divided into four groups based on the presence or absence of periodontitis (PE) and probiotic treatment (PROB): MS (no PE, no PROB), MSP (no PE, with PROB), MSPE (with PE, no PROB), and MSPEP (with PE, with PROB). The probiotic strain *Bifidobacterium animalis* subsp. *lactis* HN019 was administered beginning in week 8, while periodontitis was induced at week 14 via ligature placement around the lower first molars. Euthanasia was performed at week 16, followed by biomolecular, immunoenzymatic, and histomorphometric analyses. The results showed a significant reduction in alveolar bone loss in the probiotic-treated groups compared to the controls [58]. In another experimental study, 32 rats were assigned to four groups: control (C), probiotic-treated control (C-HN019), experimental periodontitis (EP), and EP with probiotic treatment (EP-HN019). From day 0, rats in the C-HN019 and EP-HN019 groups received daily oral administration of *B. animalis* subsp. *lactis* HN019 at a concentration of 1×10⁹ CFU/mL. On day 14, periodontitis was induced in the EP and EP-HN019 groups by placing a silk ligature around the mandibular molars. The study evaluated the effects of systemic administration of *Bifidobacterium* following ligature placement. All animals were euthanized on day 28. Oral biofilm, gingival tissues, blood serum, and mandibular bone samples were collected for microtomographic, histomorphometric, microbiological, and immunological analyses. Statistical evaluation was conducted with a significance level set at p<0.05. The findings demonstrated that the administration of *Bifidobacterium lactis* HN019 conferred protection against alveolar bone loss, likely through modulation of local and systemic microbiological and immunoinflammatory responses [59]. Furthermore, Lucateli et al. indicated that the bone-preserving effect of probiotics in animal models was strain-dependent [60].

Preclinical studies also support the protective effect of the probiotic *B. animalis* subsp. *lactis* HN019 (HN019) against periodontitis in rats undergoing chemotherapy. Eighty male rats were randomly allocated into experimental groups to receive different combinations of 5-fluorouracil (5-FU) chemotherapy, probiotics, and ligature-induced periodontitis. Relative to the chemotherapy-only group, animals administered probiotics exhibited attenuated alveolar bone loss and greater preservation of periodontal connective tissue. Probiotic supplementation also led to increased bone volume and modulated immune responses, as evidenced by reduced expression of bone-resorptive enzymes and pro-inflammatory cytokines, along with elevated osteoprotegerin levels. Microbial analysis of fecal samples revealed an increased abundance of *B. lactis* and a concomitant reduction in pathogenic oral bacteria. These findings suggest that *B. lactis* HN019 may attenuate the severity of periodontitis in the context of chemotherapy, potentially through the modulation of inflammatory pathways and inhibition of biofilm-forming pathogens [61].

The ability of probiotics to regenerate periodontal tissue was also observed through the application of reuterin in both periodontal ligament stem cells (PDLSCs) in vitro and a rat model of experimental periodontitis. Various assays - including scratch wound migration, real-time polymerase chain reaction (PCR), alkaline phosphatase activity, Alizarin red staining, RNA sequencing, and Western blot - were performed to assess reuterin’s effects on cell proliferation, osteogenic differentiation, and inflammation control. Reuterin treatment reduced TNF-α-induced endoplasmic reticulum (ER) stress in PDLSCs, restoring their regenerative functions by inhibiting intercellular ER stress transmission via connexin 43. In vivo, reuterin enhanced periodontal tissue regeneration and decreased local inflammation, suggesting its potential as a therapeutic agent for periodontitis [56].

Together, these preclinical studies highlight the promise of specific probiotic strains and their metabolites as adjunctive therapies for preserving bone, modulating inflammation, and supporting tissue regeneration in periodontal disease (Table 1).

**Table 1:** Preclinical Evidence on Probiotics in Periodontal Disease. Abbreviations: EP, Experimental periodontitis; DM, diabetes mellitus; MS, metabolic syndrome; PE, periodontitis; OVX, ovariectomized; Prob, probiotic ; Met, metformin; 5FU, 5-fluorouracil; PDLSCs, periodontal ligament stem cells; ER stress, endoplasmic reticulum stress; IL-1β, interleukin-1 beta; TNF-α, tumor necrosis factor-alpha; Cx43, connexin 43; *L. reuteri*, *Lactobacillus reuteri*; *L. casei* 01, *Lacticaseibacillus casei* 01; *L. rhamnosus* EM1107, *Lactobacillus rhamnosus* EM1107; HN019, *Bifidobacterium animalis* subsp. *lactis* HN019.

Author (Year)	Model	Intervention	Comparator	Outcomes	Key Findings
Silva et al. (2023) [57]	Diabetic rats with ligature-induced EP	*L. rhamnosus* EM1107 alone or with Met	(1) Control, (2) EP, (3) EP+DM, (4) EP+Prob, (5) EP+DM+Prob, (6) EP+DM+Prob+Met	Metabolic, inflammatory, oxidative stress, and bone remodeling parameters.	*L. rhamnosus* EM1107 reduced IL-1β, TNF-α, and inflammatory infiltrate, lowered blood glucose, and decreased bone loss; effects were enhanced when combined with Met
Moreira et al. (2023) [58]	Rats with MS (high-fat diet) with EP	Systemic administration of *Bifidobacterium animalis* subsp. *lactis* HN019	4 groups based on presence of MS, PE, and Pro treatment	Alveolar bone loss; Hepatic steatosis and proteinuria; Intestinal morphology and microbiota; Adipose tissue lipogenic gene expression	HN019 reduced alveolar bone loss, improved hepatic and intestinal parameters, and downregulated lipogenic genes in adipose tissue, indicating systemic and local protective effects
de Oliveira et al. (2023) [59]	Ligature-induced EP in rats	Daily systemic administration of Bifidobacterium animalis subsp. lactis HN019	EP group (untreated)	Alveolar bone loss (microtomographic, histomorphometric); Immunoinflammatory markers); Oral biofilm composition	HN019 reduced alveolar bone loss by modulating immunoinflammatory and altering oral biofilm composition
Lucateli et al. (2024) [60]	OVX rat model of osteoporosis.	Oral *B. animalis* subsp. *lactis* HN019 or *L. casei* 01 for 4 months	C-OVX (control), OVX-HN019 and OVX-LC01	Alveolar bone porosity, connective tissue density; Intestinal morphology and barrier markers; Blood estradiol	Both Pro strains reduced alveolar bone destruction, improved intestinal barrier, and increased estradiol; *L. casei* 01 showed greater effectiveness than HN019, indicating strain-dependent effects
Maia et al. (2023) [61]	EP in rats, with or without 5-FU chemotherapy	Daily administration of *Bifidobacterium animalis* subsp. *lactis* HN019	EP-5FU group (diseased + chemotherapy) and EP group (diseased only)	Bone/connective tissue loss, bone volume (morphometric, histomorphometric, microtomographic); Immunoinflammatory markers Microbiological markers	HN019 reduced periodontitis severity in rats undergoing chemotherapy by: Modulating immunoinflammatory parameters and Reducing periodontopathogen expression in the biofilm
Han et al. (2024)[56]	Ra twith EP; PDLSCs in vitro	Local injection of reuterin (a bioactive isolated from *L. reuteri*)	Untreated control/TNF-alpha-stimulated cells	PDLSC proliferation, osteogenic differentiation, ER stress, tissue regeneration, inflammation	Reuterin enhanced periodontal tissue regeneration, reduced inflammation, and restored PDLSC function by inhibiting Cx43-mediated ER stress.

Clinical evidence

Probiotics as an Adjuvant to SRP

Probiotics, particularly *L. reuteri,* have been assessed in terms of periodontal health, as shown by Ho et al. based on the assessment of ten randomized controlled trials (RCTs) in 430 patients through systematic review and meta-analysis, four studies reported a statistically substantial improvement in clinical attachment level (CAL) among individuals taking probiotics, while the other six studies found no difference between the two groups. In this case, two studies also demonstrate a much greater improvement in the CAL values of deep periodontal pockets. In one-year study periods, in comparing moderate (4-6 mm) versus deep (≥6 mm) pockets, probing pocket depth (PPD) reduction was significantly greater with probiotics in three studies, while two studies found no significant difference. In the probiotic group, a smaller reduction in PPD was reported at three months in moderate pockets within the probiotic group in one study (P=.016). However, definitive conclusions are hindered by the heterogeneity of evaluation methods and the lack of supporting microbiological or immunological data [2].

Further insights were offered by Ausenda et al., who reviewed 25 studies involving 894 participants - 451 receiving probiotics and 443 assigned to control interventions. The assessment was done based on clinical parameters; from these studies, 10 also had microbiological findings and four showed immunological results. In the case of probiotics administered twice daily as lozenges and including the strain* L. reuteri,* the PPD was significantly different in the probiotic group. The difference was statistically significant in the short and medium term (p<0.05) for the gain in CAL. However, heterogeneity between studies prevented a meta-analysis of both the immunological and microbiological results [62]. Furthermore, Ochôa et al. included nine trials of 287 adults aged 18-56 years to determine the effects of *L. reuteri* as an adjunct to non-surgical periodontal treatment (NSPT). Six studies noted a definite benefit from the probiotic, while three studies did not see differences. Although baseline results were comparable, at follow-up there were significant differences between the probiotic group and the comparison group: all clinical variables were improved (p=0.001). However, there was considerable heterogeneity in terms of study design and follow-up period (between 14 and 360 days), so the authors cautioned against concluding this finding [63].

Ram et al. subjected 11 RCTs (containing a total of 369 patients) on the effectiveness of a probiotic, *L. reuteri*, in the treatment of chronic periodontitis for a systematic review where PPD reduction in eight studies was observed in a probiotics group and seven studies demonstrated significant improvements in CAL. Conversely, no statistically significant difference in PPD between the probiotic and control groups, and no difference in CAL between the two groups was observed in four studies [64]. Consistent with this systematic review restriction, evidence suggests the adjunctive application of *L. reuteri* as a complement to SRP as far as it can provide some incremental benefit in improving periodontal outcomes. Robo et al. analyzed 23 studies to evaluate probiotic use as part of NSPT for chronic periodontitis. Probiotics may be beneficial for decreasing localized inflammation and improving periodontal prognosis; however, since the studies showed variation in dosage and follow-up periods, it is unlikely that specific benefits will be observed [65]. Finally, Sachelarie et al. conducted a pilot study in which 80 patients with periodontitis were assessed with traditional NSPT and NSPT with *L. reuteri* probiotics. The probiotics group at eight weeks showed notable decreases in pathogenic bacteria, IL‑1β (-37%), TNF‑α (-42%), and several clinical measures, including a 2-mm decrease in PPD (p<0.01) as well as declines in the Gingival Index (GI, -1.5) and Bleeding Index (BI, -1.3). This suggests that probiotics may improve periodontal therapy by reducing inflammation and establishing microbial balance [55].

These findings collectively suggest that probiotics, especially *L. reuteri,* offer promising adjunctive benefits in periodontal therapy, but the overall evidence is of poor quality, with the complexity of the oral microbiome making clinicians cautious about the incorporation of probiotics in routine periodontal treatment. Well-designed, long-term RCTs with clear-cut protocols are needed to define their role and establish evidence-based guidelines [2,55,62-65].

Evaluating Probiotics as Adjuncts and Alternatives in Periodontal Therapy

Probiotics, tetracycline fibers, chlorhexidine gel, and systemic antibiotics have varying advantages and disadvantages in the treatment of periodontal disease. The more recent interest in probiotics (e.g.,* L. reuteri*) is due to their ability to significantly decrease gingival inflammation and Plaque Index (PI) clinically and without any of the negative side effects of current antibiotics [66,67].

Another RCT in 2022 by Ramos et al. studied 45 patients over 90 days and similarly found a clinical improvement in all groups: control, SRP alone, antibiotics (metronidazole and amoxicillin), and probiotics (*L. reuteri*), but the antibiotics group had greater reductions in bleeding on probing (BoP) and PPD in deep pockets (≥6 mm) with more adverse effects like headache and gastrointestinal discomfort. However, the probiotics group achieved significantly better plaque control and the fewest side effects; thus, it was considered to be the group that has the greatest potential for long-term safe use [66].

Consistent with earlier findings, a 2024 RCT by Kumararama et al. found that antibiotics produced an early reduction of PPD and BoP at one month. However, by three months, the subgroup receiving probiotics - despite having a higher PI - maintained their reduction in BoP and achieved better long-term outcomes, with no antibiotic-related adverse events [68].

Manas et al. also conducted a four-week, cross-sectional study in 60 patients with chronic periodontitis to compare tetracycline fibers, chlorhexidine gel, and probiotic mouthwash. Both tetracycline and chlorhexidine groups showed significant PPD reduction, while the probiotic mouthwash yielded the greatest improvements in PI and GI. All three treatments offered comparable overall clinical benefits, but the probiotic mouthwash stood out as an effective, non-antibiotic alternative [67]. Moreover, Puzhankara et al. reported in a systematic review of 10 clinical trials that probiotics are better than antibiotics at significantly improving CAL, yet antibiotics appear to be better at reducing PI and GI. However, the use of probiotics and antibiotics together had statistically significantly better clinical results than either of the individual drugs did on its own [69].

Overall, the evidence base for probiotic use developed through RCTs and systematic reviews is still limited due to variability in experimental designs, antibiotic types, and measurement methods [69]. The long-term benefits of probiotics, together with reduced antibiotic use, remain available, but combined therapeutic approaches demonstrate better potential for total recovery through expanded research-based treatment plans and longer-term effect understanding.

Probiotics as Local Agents

Many clinical trials provide evidence that supports the adjunctive use of probiotics in periodontal treatment, particularly for NSPT, demonstrating favorable outcomes in both clinical and microbiological parameters [70,71]. Used as local agents, the application of probiotics alongside SRP results in a significant reduction in PPD and other clinical parameters, including BoP and PI, compared to SRP alone [70,72]. When administered via various formulations such as mouth rinses, lozenges, subgingival pastes, toothpaste, and chewing gum, probiotics provided flexibility in application and greater patient acceptance [73,74].

A double-blind, randomized, placebo-controlled trial involving 40 patients with chronic periodontitis treated with *Lactobacillus brevis* and *Lactobacillus plantarum* strains administered as a gel into periodontal pockets and as lozenges showed that both the control and probiotic groups improved equally in clinical parameters. Nevertheless, logistic regression analysis indicated that probiotics significantly decreased gingival bleeding (odds ratio (OR)=2.12, p=0.048), but concurrently, increased the number of remaining diseased sites (OR=0.51, p<0.001), thereby suggesting that their adjunctive application might not be warranted [75]. In another randomized prospective study on 80 patients with periodontitis, Minić et al. indicated greater reductions in PPD (1.42-1.81 mm) in the SRP+topical probiotics group compared to SRP alone (0.38-1.22 mm) in favor of enhanced efficacy of NSPT [70]. The use of probiotic mouth rinse and chlorhexidine was compared in a double-blind randomized clinical trial involving 14 patients conducted by Chawla et al. where the probiotics-treated group showed more significant improvements in GI and microbial reduction data [71]. In addition, Butera et al. conducted an RCT that compared chlorhexidine toothpaste to two probiotic formulations in 60 patients. Both probiotic groups exhibited better clinical and microbiological results over the six months than the chlorhexidine group, especially the probiotic toothpaste and gum consumption participants [74].

Additionally, another clinical study by Poulose et al. enrolled 62 patients with periodontitis who were treated with either subgingival probiotic paste in combination with SRP or SRP alone. For the treatment group, they noted more improvement in all clinical parameters with a significant reduction from baseline to day 8, with colony-forming units demonstrating the viability and efficacy of using subgingival probiotics [72]. Soluble probiotics in lozenge form also used with SRP had a greater reduction in gingival inflammation and MMP-8 levels in gingival crevicular fluid (GCF), showing potential immunomodulatory effects of probiotics [72]. Future studies will be needed to determine if the use of probiotics will be helpful in larger studies over longer periods [73].

Prevention of Periodontitis

Probiotics' potential benefits extend to the prevention of periodontal diseases, as demonstrated by Ren et al. in a study of 4577 adults from the National Health and Nutrition Examination Survey (NHANES), 2009-2014. The researchers attempted to compare the prevalence of periodontitis among the groups and to assess the effect of probiotics in the prevention of periodontitis and their relationship. The prevalence of periodontitis was lower in the probiotic group (27.83%) than in the control group (41.08%), which was significant (p<0.001). In addition, even after the effect of several confounding variables was corrected (age, sex, ethnicity, socioeconomic status, and smoking), the association remained and it was seen that the intake of probiotics was related to a 30% lower risk of periodontitis (OR=0.70; 95% CI: 0.54-0.92; p=0.01) [76]. From this data, it could be concluded that there was a strong inverse association between probiotic intake and the prevalence of periodontal disease.

In support of this, epidemiological data were provided by Alkaya et al. through a randomized, double-blind, placebo-controlled clinical trial assessing the effects of a probiotic-enriched ayran beverage on gingival health among 54 healthy university students. The participants were randomized into either a probiotic ayran group or a control ayran group for 42 days, and they ceased regular oral hygiene for five days to induce a state of experimental gingivitis. While marginal increases in inflammation were observed in both groups, the probiotics-consuming group demonstrated relatively strong effects: lower levels of dental plaque, inflammation, gingival bleeding, and levels of MMP-8 in GCF with p-values < 0.001 to 0.002 [77]. Table 2 illustrates the clinical impact of using probiotics in the treatment of periodontal disease.

**Table 2:** Clinical Evidence on Probiotics in Periodontal Disease. Abbreviations: RCTs, Randomized controlled trials; SRP, scaling and root planing; *L. reuteri*, *Lactobacillus reuteri*; CAL, clinical attachment level; PPD, probing pocket depth; GI, Gingival Index; PI, Plaque Index; OR, odds ratio.

Author (Year)	Study Design	Intervention	Comparator	Outcomes	Key Findings
Ho et al. (2020) [2]	Systematic review and meta-analysis (10 RCTs)	Probiotics+SRP	SRP alone	Clinical, immunological and microbiological parameters	While CAL gain, and PPD reduction in the probiotics group was significant at 3 months and 12 months, no significant difference was noted at 6 months and 9 months. The results are inconclusive due to high heterogeneity and lack of microbiological data.
Ausenda et al. (2023) [62]	Systematic review (25 studies)	Probiotics mainly *L. reuteri* primarily as lozenges as an adjunct to SRP	SRP alone	Clinical, immunological and microbiological parameters	Statistically significant PPD and CAL gains in the short and medium term, but heterogeneity prevented meta-analysis of microbiological/immunological data.
Ochôa et al. (2023) [63]	Systematic review (9 RCTs)	*L. reuteri* as adjunct to SRP	SRP alone	Clinical parameters	Significantly better clinical outcomes than but the conclusion must be carefully interpreted because of the heterogeneity found among the studies.
Ram et al. (2022) [64]	Systematic review (11 RCTs)	*L. reuteri* as an adjunct to SRP	SRP alone	Clinical parameters	While clinical parameters showed improvement, the risk of bias prevent definitive conclusion.
Robo et al. (2024) [65]	Systematic review (23 studies)	Probiotic as adjunct to SRP	SRP alone	Clinical parameters	Probiotics appear beneficial as an adjunct to SRP but inconsistent evidence and protocol variations limit firm conclusions.
Sachelarie et al. (2025) [55]	Pilot study (80 patients)	*L. reuteri* with SRP	SRP alone	Clinical parameters and inflammatory markers	Probiotics, as an adjunct to periodontal therapy, effectively restore the microbiota balance, reduce inflammation, and improve clinical outcomes in periodontitis.
Ramos et al. (2022) [66]	RCT (45 patients)	*L. reuteri* with SRP Antibiotics+SRP	SRP alone	Clinical and immunological parameters	After three months, neither antibiotic nor probiotic adjuncts delivered additional benefit in terms of PDD beyond what was achieved by subgingival instrumentation alone.
Kumararama et al. (2024) [68]	RCT (60 patients)	Probiotics+SRP Antibiotics+SRP	SRP alone	Clinical parameters	Antibiotics and probiotics both improved peri-implant mucositis; antibiotics showed a more immediate effect, while probiotics provided longer-lasting benefits.
Manas et al. (2024 )[67]	Longitudinal RCT (60 patient)	Tetracycline fiber+SRP Chlorhexidine gel+SRP Probiotic rinse+SRP	SRP alone	Clinical parameters	Tetracycline fibers, Chlorhexidine gel, and probiotic mouthwash are all similarly effective for treating chronic periodontitis, but they are all more effective than SRP alone.
Puzhankara et al. (2024) [69]	Systematic review (10 clinical trials)	Probiotics as adjuvant to SRP	Antibiotics	Clinical and microbiological parameters	Probiotics showed a significant reduction in the PPD and CAL compared to antibiotics. Antibiotics were more effective in reducing the PI and GI.
Minić et al. (2022) [70]	Randomized prospective study (80 patients)	Locally applied probiotics+SRP	SRP alone	Clinical parameters	Topical application of probiotics in combination with SRP increases the effectiveness of conventional non-surgical therapy of periodontitis.
Chawla et al. (2024) [71]	Double-blind RCT (14 patients)	Probiotic mouthrinse+SRP	Chlorhexidine mouthrinse+SRP	Clinical and microbiological parameters	Both the probiotic and chlorhexidine groups achieved a significant reduction in mean clinical parameters. Regarding microbial load, chlorhexidine demonstrated a greater reduction than the probiotic group.
Butera et al. (2020) [74]	RCT (60 patients)	SRP+probiotics-based toothpaste. SRP+probiotics-based toothpaste+probiotics-based chewing gum	SRP+chlorhexidine-based toothpaste	Clinical and microbiological parameters	Topical probiotics (toothpaste and chewing gum) are a valid and effective adjunct to SRP, yielding a significant and sustained improvement in clinical parameters and reducing specific periodontopathogens.
Poulose et al. (2024) [72]	Clinical study (62 patients)	Subgingivally applied probiotics	SRP alone	Clinical and microbiological parameters	Subgingival probiotics plus SRP improved clinical outcomes in periodontitis, with bacteria remaining viable up to 8 days, making it an effective and cost-efficient adjunct therapy.
Ren et al. (2023) [76]	Observational study (NHANES survey)	Probiotic intake	Control	Prevalence of periodontitis	Probiotic intake was associated with a 30% lower risk of periodontitis (OR=0.70).
Alkaya et al. (2024) [77]	RCT (54 healthy students)	Probiotic-enriched ayran	Control ayran	Clinical and biochemical parameters	Daily consumption of a probiotic ayran drink containing *Lactobacillus acidophilus* and *Bifidobacterium bifidum* statistically significantly lowers clinical and immunological markers of gingival inflammation.

Challenges and limitations

As supportive evidence for the use of probiotics grows toward a beneficial role in periodontal therapy, several limitations remain. The strain-specific effects, dosage regimen, safety profile, especially in certain patients, and absence of large-scale, long-term RCTs to definitively establish their efficacy should be carefully considered. Patients’ adherence and acceptance across various populations also have to be assessed in future research [24,31,78].

Future directions

Despite promising preclinical and clinical findings, several research gaps must be addressed to fully establish the role of probiotics in periodontal therapy. High-quality, large-scale RCTs with extended follow-up periods are particularly needed, as current evidence is often limited by small sample sizes, short durations, and methodological heterogeneity (62,63,65). Future studies should standardize protocols, establish optimal dosages and frequencies, and assess long-term efficacy to ensure sustainable periodontal benefits.

Furthermore, evidence consistently indicates that probiotic effects are strain-dependent. Future research should systematically compare different strains and their effects on specific pathogens and host inflammatory responses. In addition, investigating synbiotics - a combination of probiotics and prebiotics - could enhance colonization and therapeutic effects in the oral cavity. Moreover, combined probiotic and antibiotic therapies warrant further exploration, as preliminary studies suggest superior outcomes by simultaneously reducing pathogenic load and restoring microbial balance [69].

Another important avenue is mechanistic research. Advanced techniques such as metagenomics and metabolomics could identify biomarkers to predict patient responses to probiotic therapy, while studies of the gut-oral microbiome axis may reveal systemic effects of probiotics on periodontal health. Finally, preventive applications of probiotics in at-risk populations should be explored, as early evidence indicates a potential role in reducing periodontal disease prevalence [76,77]. By addressing these avenues, the field can move from promising evidence toward evidence-based clinical guidelines for the use of probiotics in both prevention and management of periodontal diseases.

## Conclusions

Accumulating preclinical and clinical evidence suggests that probiotics may play a valuable role in the management of periodontal diseases. Their beneficial effects stem from their ability to modulate the oral microbiome and bone metabolism, mitigate inflammatory responses, and protect periodontal tissues, which collectively lead to improved clinical outcomes.

Despite these encouraging findings, significant challenges persist. The lack of standardized protocols, strain-specific variability, safety concerns, and limited long-term data from large-scale trials constrain their widespread adoption in routine practice. Addressing these limitations through rigorous, well-designed studies is essential to define the precise role of probiotics as adjunctive agents in the management of periodontal disease.
